# Self-Deception in Addiction Rehabilitation: Impulsivity and Self-Efficacy as Predictors of Manipulation and Mystification

**DOI:** 10.3390/bs16030456

**Published:** 2026-03-19

**Authors:** Javier Sampedro, Llanos Merín, Laura Ros, Jorge J. Ricarte

**Affiliations:** Department of Psychology, University of Castilla-La Mancha, 02071 Albacete, Spainlaura.ros@uclm.es (L.R.); jorgejavier.ricarte@uclm.es (J.J.R.)

**Keywords:** substance use disorder, self-deception, impulsivity, self-efficacy, addiction rehabilitation

## Abstract

Substance use disorder (SUD) is a global problem with serious psychological, physiological, and social consequences. Seeking professional help is often delayed due to a lack of self-recognition of addiction, frequently sustained by self-deception. Although self-deception is a core feature of SUD, the mechanisms underlying it remain insufficiently studied. This research examines the role of impulsivity and self-efficacy in predicting self-deceptive behaviors—manipulation and mystification—in individuals undergoing addiction rehabilitation. The sample consisted of 122 Spanish participants from therapeutic communities (Mage = 44.99, SD = 10.58; 82.8% male) who completed the Self-Deception Questionnaire (SDQ-12), the Impulsive Control Scale Ramón y Cajal (ECIRyC), and the Drug Taking Confidence Questionnaire (DTCQ). Results showed that impulsivity significantly predicted both manipulation and mystification. Manipulation was also associated with self-efficacy in managing temptation and duration of addiction, highlighting its multifaceted nature. In contrast, mystification was predicted solely by impulsivity, suggesting a stronger dependence on internal psychological processes rather than contextual factors. These findings underscore the importance of targeting impulsivity and enhancing self-efficacy in addiction treatment. Interventions such as Cognitive Behavioral Therapy (CBT), Acceptance and Commitment Therapy (ACT), and mindfulness-based approaches may be particularly effective in reducing self-deceptive behaviors and supporting long-term recovery.

## 1. Introduction

Substance use disorder (SUD) is a global psychological problem. According to the latest World Health Organization (WHO) report, worldwide in 2019 there were 2.5 billion people above 15 years old with alcohol use disorder, and, in 2021, approximately 296 million people aged 15–64 years used psychoactive drugs (World Health Organization, 2024). These conditions can have serious physiological, psychological, and social consequences, not only for substance users themselves but often also for those around them (McNally et al., 2023). Thus, identifying predictors for treatment success in patients with SUD is of major interest in addiction research.

There is a broad agreement that individuals with SUD often exhibit deceptive behaviors, as it is commonly conceptualized as a disorder marked by self-delusion, denial, mystification, and dishonesty (Latkin et al., 2017). Therefore, a crucial component of effective treatment involves addressing the individual’s denial, self-deception, and resistance to change (Ferrari et al., 2008). However, the variables that maintain deceptive tendencies have been rarely studied within drug-addicted populations in treatment, despite their potential significance in facilitating recovery, preventing relapses, and contributing to treatment success (Caputo, 2019; Marlatt & Donovan, 2005). Although self-deception is widely acknowledged as a defining feature of addiction, considerably less attention has been given to the psychological mechanisms that may sustain or intensify these distortions during rehabilitation (Caputo, 2019; Ferrari et al., 2008). A clearer understanding of these mechanisms is necessary to explain why some individuals persist in defensive or manipulative patterns despite being in treatment. In this context, impulsivity and self-efficacy emerge as theoretically relevant candidates, as they represent complementary processes related to self-regulation and coping in addiction (Stevens et al., 2014; Kadden & Litt, 2011).

This study aims to examine such self-deceptive behaviors and identify potential predictors, including impulsivity and self-efficacy, in a sample of people with SUD following rehabilitation.

Self-deception: Manipulation and Mystification

Lying and self-deception are considered intrinsic aspects of the human condition and play a regulatory role in psychological protection (Martínez-González et al., 2016). Self-deception results from cognitive and social biases in information processing, which prioritize favorable over unfavorable information, either consciously or unconsciously, in alignment with an individual’s goal (Von Hippel & Trivers, 2011). Properly used, self-deception is associated with various positive psychological and social outcomes, including an individual’s sense of happiness, self-esteem, self-confidence, emotional memory, social cognition, and altruistic behavior (Lopez & Fuxjager, 2012; McKay et al., 2005; Ren et al., 2018). In contrast, pathological self-deception refers to a form of self-deception where individuals are unable to acknowledge the negative consequences of their behavior or, despite being aware of the problem, fail to adopt solutions, refuse to do so, or wait for these solutions to come elsewhere (Sirvent et al., 2011). Substance-dependent patients show higher pathological self-deception than their non-dependent peers, with manipulation and mystification being some of the mechanisms of self-deception that best describe substance-dependent patients (Moral & Sirvent, 2014).

Manipulation is defined as the intentional and strategic effort to influence others’ emotional states or behaviors to achieve personal objectives, usually without respecting their needs or feelings (Khedr et al., 2023). Individuals with SUD frequently rely on manipulative strategies to sustain their substance use, targeting those within their family and social circle. Commonly used tactics of manipulation in this population include making empty promises, adopting a victimized personality to elicit sympathy, offering excuses for their irresponsibility, inducing guilt or discomfort in others to meet unreasonable demands, and, in more extreme cases, threatening self-harm as a coercive strategy (Caputo, 2019). Moreover, manipulation often involves a deliberate mystification process aimed at achieving self-serving objectives (Moral & Sirvent, 2014).

Clinical mystification refers to a marked disconnection from reality that disrupts the individual’s vital activities and overall development (Ziegler et al., 2012). It creates a psychological barrier characterized by distrust and the recurrent use of denial mechanisms, which impede effective interpersonal communication and serve to distort reality and maintain self-protective delusions (Sirvent et al., 2011). In more extreme cases, clinical mystification can evolve into what has been described as “deception as a way of life”, a state that includes the adoption of a false external appearance and even a misanthropic attitude (Sirvent et al., 2019). Both manipulation and mystification not only sustain maladaptive behaviors but also hinder therapeutic progress by promoting mistrust and impeding meaningful communication (Moral & Sirvent, 2014).

Within the context of addiction, these two dimensions reflect distinct but related processes, suggesting that self-deception in SUD is not a unitary construct. Manipulation refers to outward-oriented strategies aimed at influencing others to maintain substance use or avoid responsibility, whereas mystification reflects a more internalized distortion of reality characterized by denial, rationalization, and self-delusion. Differentiating these dimensions is clinically relevant, as they may be sustained by partially different psychological mechanisms and may therefore require differentiated intervention strategies (Sirvent et al., 2011; Moral & Sirvent, 2014). However, despite their clinical relevance, little empirical research has examined which psychological factors may predict manipulation and mystification during rehabilitation (Caputo, 2019).

Impulsivity and the Maintenance of Self-Deceptive Processes in Addiction

Impulsivity is one potentially relevant predictor of drug-related behaviors that remains relatively understudied (Jentsch et al., 2014; Sliedrecht et al., 2021). It is a multidimensional construct defined as the inability to stop behaviors with negative consequences, preference for immediate over delayed gratification, tendency towards risky actions, heightened desire for novelty, acting without consideration of outcomes, impatience when asked to wait, short attention span, and difficulty maintaining focus on a specific task (Perry & Carroll, 2008).

Impulsivity plays a significant role in the development and maintenance of addiction, affecting various stages, from the initial use of substances to relapse after periods of abstinence (Vassileva & Conrod, 2019).

Stevens et al. (2014) identified two aspects of impulsivity in patients with SUD: impulsive choice (i.e., the preference for immediate reward over delayed ones) and impulsive action (i.e., the difficulty in inhibiting responses, such as resisting the urge to use substances despite knowing the risks). Both impulsive choice and action interact to exacerbate addiction by impairing self-regulation and increasing susceptibility to cravings and relapses (Sliedrecht et al., 2021). Importantly, deficits in self-regulation associated with impulsivity may not only increase substance use directly but may also foster cognitive distortions aimed at protecting immediate reward-seeking behavior (Dawe & Loxton, 2004; Stevens et al., 2014).

Impulsive individuals typically exhibit diminished inhibitory control and heightened sensitivity to reward, which compromise executive functioning and long-term decision-making processes (Bechara et al., 2013; Vassileva & Conrod, 2019). As a consequence, when short-term relief or gratification is prioritized over long-term consequences, individuals may experience cognitive dissonance between their behavior and their awareness of its negative outcomes. To reduce this psychological discomfort, they may engage in denial, rationalization, and minimization processes that reframe substance use in a less threatening manner (Martínez-González et al., 2016; Von Hippel & Trivers, 2011).

From this perspective, self-deception may operate as a regulatory strategy that allows impulsive individuals to maintain reward-driven behavior while protecting their self-concept and reducing awareness of loss of control (Dawe & Loxton, 2004; Sirvent et al., 2011). Therefore, impulsive individuals may be particularly susceptible to constructing self-deceptive justifications for their behavior, including minimizing the dangers associated with drug use or distorting their perceived capacity to control consumption (Dawe & Loxton, 2004; Moral & Sirvent, 2014).

Self-efficacy and Self-Deceptive Processes in Addiction

Self-efficacy refers to a person’s confidence to perform the behaviors necessary to achieve a desired goal (Mokuolu & Ajiboye, 2020). According to previous studies, self-efficacy plays a role in both initiation and cessation of substance use. Low self-efficacy can significantly increase the risk of substance use, as individuals may feel powerless against peer pressure, stress, or temptation situations, often resorting to drug use as a coping mechanism (Kadden & Litt, 2011; Soravia et al., 2015). Additionally, it can create a vicious cycle where the belief in one’s inability to control risky situations for substance use leads to continued or escalating behaviors, which in turn reduces self-efficacy (Fathiandastgerdi et al., 2016). In contrast, high self-efficacy might help individuals resist the temptation of using substances by enabling them to manage stress and negative emotions (Yang et al., 2019). Moreover, those with stronger self-efficacy are more likely to choose environments and behaviors that reduce the probability of substance use (Mokuolu & Ajiboye, 2020).

Within addiction treatment, self-efficacy is therefore conceptualized as a central regulatory resource that shapes how individuals interpret risk situations, manage craving, and respond to emotional distress (Kadden & Litt, 2011). According to social cognitive theory, perceived efficacy influences not only behavioral persistence but also cognitive appraisal processes, including how individuals interpret responsibility and control over their actions (Bandura, 1997).

These cognitive appraisal processes may be directly linked to self-deceptive mechanisms. When individuals perceive themselves as incapable of coping effectively with high-risk situations, defensive cognitive strategies, such as denial, minimization, or externalization of responsibility, may serve as compensatory mechanisms that protect perceived competence and reduce anxiety (Fathiandastgerdi et al., 2016; Yang et al., 2019; Fan et al., 2020). In this way, low self-efficacy may indirectly facilitate self-deceptive processes. Conversely, stronger self-efficacy has been associated with more active coping, greater emotional regulation to confront risk situations and tolerate the discomfort, and lower vulnerability to maladaptive avoidance (Kadden & Litt, 2011; Yang et al., 2019). When individuals feel capable of managing temptation and distress, the need to justify substance use through distortion, denial, or manipulation may diminish.

Although self-efficacy is widely recognized as a determinant of health behavior change, its specific role in the emergence and maintenance of self-deceptive processes in SUD remains insufficiently examined (Fathiandastgerdi et al., 2016; Mokuolu & Ajiboye, 2020). Clarifying this relationship is essential for understanding how perceived regulatory capacity may either mitigate or intensify defensive distortions during rehabilitation.

The Current Study

The literature suggests that, while self-deception can act as an immediate coping mechanism for individuals with addictive behaviors, it can undermine the recovery process by preventing them from confronting the underlying causes and consequences of their addiction (Moral & Sirvent, 2014). Moreover, impulsivity and self-efficacy can be integrated within a self-regulation framework to explain self-deceptive processes in addiction. Impulsivity reflects deficits in inhibitory control and heightened reward sensitivity that favor immediate gratification, whereas self-efficacy represents a perceived regulatory resource that shapes risk appraisal and coping capacity (Bandura, 1997; Bechara et al., 2013; Kadden & Litt, 2011; Stevens et al., 2014).

Understanding how factors like impulsivity and self-efficacy may influence cognitive distortions and self-justifications that perpetuate maladaptive behaviors related to self-deception may contribute to more effective rehabilitation outcomes. Based on this, this study aims at examining self-deceptive dynamics in a sample of participants with a history of addiction following rehabilitation, focusing on the roles of impulsivity and self-efficacy. Specifically, two research goals are addressed: (1) to investigate the associations between self-deception, impulsivity, self-efficacy, and addiction history; and (2) to explore which variables significantly predict manipulation and mystification in the context of addiction.

## 2. Materials and Methods

### 2.1. Participants

Participants in our study were recruited within different therapeutic communities for drug rehabilitation treatment. Inclusion criteria were: (1) participants aged between 18 and 65 years, (2) being diagnosed with SUD, (3) following a rehabilitation program, with a situation of abstinence at the time of incorporation into the study, and (4) having signed informed consent. The final sample comprised 122 participants (Mage = 44.99, SD = 10.58, 82.8% males). Regarding their story of drug misuse, 54.1% of them had initiated drug use before the age of 18 (M = 19.79, SD = 7.98), being the average duration of the addiction 16.28 years (range: 1–48 years); 43.4% of participants used alcohol as the primary substance, followed by cocaine (40.2%), cannabis (8.2%), heroine (6.6%), benzodiazepines (0.8%) and gambling (0.8%). About treatment-related variables, 82% of participants had already received previous treatments, and 44.3% presented another diagnosed psychopathology.

### 2.2. Measures

Background information. Sociodemographic characteristics (gender, age, educational level, occupation, family or marital status, and presence of other psychopathology or illnesses), history of drug problematic use (first drug use, type of primary substance, and years of sustained addiction), and number of previous treatments were assessed.

Self-Deception Questionnaire (SDQ-12; Sirvent et al., 2019). The SDQ-12 is a 12-item questionnaire that measures two dimensions of self-deception: manipulation and mystification. Each subscale is composed of 6 items, each with responses adhering to a 5-point Likert scale (from 1, strongly disagree, to 5, strongly agree). Manipulation measures the respondents’ self-presentation with the intention of influencing others’ behavior (e.g., I often choose to answer not with the truth but rather with whatever is most convenient for me). Mystification refers to the respondents’ distance from reality, in terms of not being aware of important aspects of their lives (e.g., It take me a while to become aware of certain key issues in my life), inaccurate perceptions (e.g., In important personal matters in my life, I think I repeatedly make the same mistakes), and distortion about one’s lifestyle (e.g., I feel my lifestyle is a sham. I live a lie). Scores for each subscale can range from 1 to 30. Internal consistency for the SDQ-12 is adequate, with a Cronbach’s alpha coefficient of 0.85.

Impulsive Control Scale Ramón y Cajal (ECIRyC; Ramos-Brieva et al., 2002). Comprising 20 items, this scale assesses impulse control through different behavioral manifestations of impulsivity such as lack of control and planning, intransigence, unpredictability of consequences, inability to delay gratification, and disregard of risk. The ECIRyC consists of four specific dimensions (impulsivity, immediacy, imposition, and risk) and another unspecified dimension with only one item. It is evaluated through a Likert scale of 4 points: always, often, rarely, and never. Cronbach’s alpha for the full scale was 0.84 in the validation study by Ramos-Brieva et al. (2002). Cronbach’s alpha in our sample was 0.85, indicating good internal consistency.

Drug Taking Confidence Questionnaire (DTCQ, León, 2001). Originally developed by Annis and Martin (1985), the DTCQ evaluates the participant’s level of self-efficacy in being able to cope with potential high-risk situations for substance use. The DTCQ is a 60-item self-report questionnaire consisting of a 6-point Likert scale ranging from 0 (not at all confident) to 100 (very confident). Participants respond in terms of a particular substance of use. The questionnaire is divided into eight subscales, which in turn can be grouped into three main factors: (1) Positive Situations (pleasant times with others and pleasant emotions), (2) Negative Situations (unpleasant emotions, physical discomfort and conflict with others), and (3) Temptation Situations (urges and temptations, social pressure and testing personal control). Cronbach’s alphas for items in the three factors are 0.74, 0.61, and 0.88, respectively.

### 2.3. Procedure

The Clinical Research Ethics Committee approved the study protocol (reference number: Record No. 955), which was also approved by the coordinators of the participating therapeutic communities. We held meetings with the staff of the different drug rehabilitation centers and with the potential participants, in order to present our aims, respond to any doubts or suggestions, and give them the informed consent forms for participation. Data collection was carried out within the facilities of the treatment centers by the main researcher, and all questionnaires were administered collectively in a room reserved for this purpose. The participants received a prior explanation of the different questionnaires to facilitate their understanding, and the researcher was present throughout the session to answer any questions. Evaluations occurred over one session of around 45 min.

### 2.4. Data Analysis

All analyses employed IBM SPSS Statistics 28 (SPSS, Inc., Chicago, IL, USA), and the criteria for statistical significance were set at *p* < 0.05. First, we checked the variables followed a normal distribution, using the Kolmogorov-Smirnov test and graphical procedures. Second, preliminary descriptive statistics and Pearson’s correlations among the main study variables were calculated. Finally, we performed two stepwise regression models to identify which variables could significantly predict outcomes in the two domains of self-deception, that is, manipulation and mystification. A forward stepwise regression approach was used as an exploratory method to determine which variables contributed uniquely to manipulation and mystification among several theoretically relevant and partially correlated predictors. For the main analysis, the following variables were used: years of sustained addiction, number of previous treatments, manipulation and mystification from the SD Q, total impulsivity score from the ECIRyC, and the three main factors of the DTCQ (Positive Situations, Negative Situations, and Temptation Situations).

## 3. Results

Table 1 shows the descriptive results of the study variables, while Table 2 presents the bivariate correlations.

Overall, some small-to-medium-sized correlations were detected among the examined variables. Both manipulation and mystification showed a positive linear association with impulsivity and a negative linear association with the Negative Situations and Temptation Situations factors of the DTCQ. The Positive Situations factor showed no significant correlations with any of the other variables. Years of sustained addiction were positively and significantly associated with the number of previous treatments, but these two variables were not related to others.

Forward stepwise linear regression was used to identify the most influential predictors of self-deception. Table 3 shows the results of the regressions with manipulation and mystification as dependent variables. In both cases, the criteria for variable entry and removal were set at a probability of F-to-enter ≤ 0.050 and a probability of F-to-remove ≥ 0.100.

For the manipulation dimension, the final model included impulsivity, self-efficacy over temptation situations, and years of addiction as significant predictors. This model demonstrated a good fit, accounting for a 28% of the variance in manipulation (F (3, 116) = 16.12, *p* < 0.001). Both impulsivity and years of addiction showed a significant positive impact, although impulsivity was shown to be more strongly related to manipulation (Adjusted R^2^ = 0.23, F (1, 118) = 16.12, *p* < 0.001). On the contrary, the temptation situations factor of the CTCQ showed a negative standardized coefficient, suggesting its contribution to reducing manipulation.

In the case of mystification, the only significant predictor was impulsivity, explaining 17% of the variability (F (1, 118) = 23.87, *p* < 0.001). Years of addiction, number of treatments, and the three dimensions of the CTCQ did not predict mystification values.

## 4. Discussion

While self-deception is widely recognized as a defining feature of addiction, the mechanisms that sustain these behaviors and their interactions with other psychological constructs like impulsivity and self-efficacy remain underexplored (Caputo, 2019; Ferrari et al., 2008). This study contributes to the understanding of these mechanisms by examining two dimensions of self-deception, manipulation and mystification. Together, these findings support a self-regulation framework in which impulsivity and perceived coping capacity play complementary roles in sustaining self-deceptive processes during addiction rehabilitation.

On one hand, manipulation involves strategic behaviors aimed at influencing others to maintain substance use or evade responsibility (Khedr et al., 2023). Our findings highlight impulsivity as a significant driver of manipulative behaviors. The positive correlation between impulsivity and manipulation supports the notion that individuals with impaired impulse control are more likely to use manipulation to fulfill immediate desires, such as obtaining substances or avoiding negative consequences (Jentsch et al., 2014; Perry & Carroll, 2008). Additionally, the regression analysis further reinforces this connection by identifying impulsivity as the strongest predictor of manipulation. As noted by Latkin et al. (2017), impulsive individuals tend to prioritize immediate rewards over long-term well-being or ethical considerations, which increases the likelihood of manipulative behaviors. These behaviors often involve exploiting social relationships for personal gain or to mitigate emotional distress, which aligns with the findings of studies on self-deception in SUD (Dawe & Loxton, 2004; Megías-Robles et al., 2023). In this sense, manipulation can be understood as an externally oriented form of self-deception, allowing individuals to maintain substance use while deflecting responsibility and protecting immediate reward-seeking behavior (Khedr et al., 2023).

In addition to impulsivity, self-efficacy in managing temptation situations emerged as a protective factor against manipulation. Self-efficacy in temptation situations not only showed a significant negative correlation with manipulation but also served as a negative predictor. Specifically, individuals with greater self-efficacy demonstrated a lower tendency to engage in manipulation, indicating that confidence in one’s ability to handle high-risk situations may reduce the reliance on manipulative behaviors. These results align with Bandura’s self-efficacy theory (Bandura, 1997), which asserts that a strong belief in one’s capabilities can lead to more adaptive coping strategies, such as the ability to manage temptations or cravings, thereby decreasing the likelihood of resorting to self-deception and manipulation (Fathiandastgerdi et al., 2016; Kadden & Litt, 2011). In support of this, Yang et al. (2019) found that individuals with higher self-efficacy in coping with temptation were also less likely to relapse. Similarly, Freire et al. (2020) emphasized that self-efficacy enhances self-regulation, enabling individuals to resist the desire for immediate rewards (such as substance use) in favor of long-term health benefits. Consequently, individuals with higher efficacy in managing risky situations tend to behave more prosocially, demonstrate fewer impulsive behaviors, and engage in healthier lifestyle choices compared to those with lower self-efficacy (Fan et al., 2020), making it less necessary to engage in self-deceptive behaviors like manipulation.

On the other hand, mystification represents a deeper and more internalized dimension of self-deception involving denial, distorted perceptions, and a disconnection from reality (Sirvent et al., 2011, 2019). This study identified impulsivity as the only significant predictor of mystification. These findings suggest that impulsivity not only drives external behavior like manipulation but also sustains internal cognitive distortions. The link between impulsivity and mystification can be explained by the tendency of impulsive individuals to seek immediate relief from psychological distress, often through cognitive avoidance strategies such as denial and rationalization (Dawe & Loxton, 2004; Whiteside & Lynam, 2001). Impulsivity impairs self-regulation, leading to emotion-driven responses, including reliance on denial and distorted perception to manage guilt, fear, or shame associated with addiction (Jentsch et al., 2014; Stevens et al., 2014). Furthermore, neurological evidence supports the association between impulsivity and mystification through dysfunctions in the prefrontal cortex, which compromises self-control and decision-making, fostering mystification as a coping mechanism (Bechara et al., 2013; Naqvi et al., 2007). Therefore, mystification serves as a coping mechanism for impulsive individuals, allowing them to rationalize their substance use and maintain a sense of psychological stability despite the consequences of addiction (Stevens et al., 2014; Ziegler et al., 2012). Unlike manipulation, mystification appears to operate as a predominantly internal regulatory mechanism, rooted in cognitive distortion and avoidance rather than interpersonal strategy.

Notably, self-efficacy is not a predictive factor for mystification. This suggests that cognitive distortions associated with mystification function independently of an individual’s confidence in managing challenging situations. It is plausible that, unlike manipulation, which is influenced by external interpersonal factors, mystification is rooted in deeper psychological processes that are less dependent on situational elements like self-efficacy (Sirvent et al., 2011; Ziegler et al., 2012). This highlights the internalized nature of mystification as a coping mechanism. However, further research is needed to fully understand these relationships (Caputo, 2019; Dawe & Loxton, 2004).

Finally, our results also showed that years of sustained addiction were positively associated with manipulation but did not predict mystification. Prolonged addiction may reinforce self-deception through justifications for harmful behaviors and the use of manipulative strategies to maintain substance use (Sirvent et al., 2011). However, the lack of association between addiction length and mystification might imply that mystification involves more complex cognitive distortions or emotional defenses that are not necessarily tied to the duration of the addiction (Ziegler et al., 2012). These findings suggest that different mechanisms may drive each form of self-deception, requiring distinct therapeutic approaches for treatment.

While this study provides valuable insights into the predictors of self-deception in addiction recovery, several limitations must be acknowledged. First, the study’s cross-sectional design limits causal inferences, as it cannot establish the directionality of the relationships between impulsivity, self-efficacy, and self-deception. Future research should employ longitudinal designs assessing these variables at treatment entry and following participants throughout therapy and after completion to determine their predictive value for recovery trajectories and relapse outcomes. Second, the reliance on self-report measures may introduce biases related to social desirability or retrospective recall, especially when participants were asked about their addiction history and self-deceptive behaviors (Latkin et al., 2017). Although addiction history was operationalized as years of sustained addiction and the number of previous treatments, future studies could incorporate additional indicators such as severity indices, relapse frequency, or treatment adherence to provide a more comprehensive characterization. Third, although the sample was predominantly male (82.8%), this distribution reflects the epidemiological reality of many residential treatment programs and therapeutic communities (World Health Organization, 2024). While gender differences have been reported in some patterns of substance use, core mechanisms involved in addiction, such as impulsivity, self-regulation, and cognitive distortion processes, appear to operate similarly across genders (Perry & Carroll, 2008; Vassileva & Conrod, 2019). Nevertheless, future studies should explicitly examine potential gender differences in manipulation, mystification, and their psychological predictors. Additionally, the diagnostic heterogeneity of the sample, with a predominance of alcohol use, may limit substance-specific interpretations. However, this clinical diversity reflects real-world treatment settings and supports the ecological validity of the study. Moreover, both self-deception and impulsivity have been conceptualized as transdiagnostic mechanisms operating across substance-related and behavioral addictions (Caputo, 2019; Dawe & Loxton, 2004; Jentsch et al., 2014). Future research should examine whether distinct patterns emerge across different substances or between substance-related and behavioral addictions in the expression of manipulation and mystification. Finally, while this study focuses on impulsivity and self-efficacy, other psychological and social factors may also play a critical role in self-deception during recovery. For instance, emotional regulation, cognitive flexibility, and social support are important components of the recovery process and could influence how individuals cope with self-deception (Caputo, 2019; McNally et al., 2023). Investigating these additional factors may provide a more comprehensive understanding of self-deceptive behaviors in addiction recovery. Additionally, the use of stepwise regression may present certain limitations. Therefore, the predictive findings should be interpreted as exploratory and replicated in future studies using confirmatory regression models and larger samples.

Despite these limitations, the findings of this study have important implications for addiction rehabilitation programs. The strong connection between impulsivity and self-deceptive behaviors suggests that interventions targeting impulse control could be key in reducing the reliance on manipulative strategies or cognitive distortions. For instance, Cognitive Behavioral Therapies (CBT) and other behavioral approaches that enhance self-regulation may be particularly effective in addressing manipulation and its consequences (Sliedrecht et al., 2021; Zamboni et al., 2021). Similarly, cognitive distortions, such as denial and rationalization, which sustain mystification, may be effectively managed through therapies like Acceptance and Commitment Therapy (ACT) or mindfulness-based interventions, which have been shown to reduce cognitive defenses (Alfonso et al., 2011; Berman & Kurlancheek, 2021). Additionally, self-efficacy was found to be a protective factor against manipulation, highlighting the importance of interventions aimed at strengthening confidence in managing temptation and high-risk situations. Programs focusing on building self-efficacy through skills training, goal-setting, and mastery experiences (Kadden & Litt, 2011; Zamboni et al., 2021) could help individuals reduce the likelihood of engaging in manipulation or substance use when confronted with challenges. Future research should expand on these findings by exploring additional factors and developing rehabilitation strategies that address the role of self-deception in recovery.

## Figures and Tables

**Table 1:** Descriptive statistics for key variables (*N =* 122).

Variable	Mean (SD)	Range
Years of addiction	16.28 (11.09)	1–48
Number of previous treatments	1.94 (1.72)	0–9
SDQ Manipulation	17.33 (6.13)	6–30
SDQ Mystification	21.47 (6.49)	6–30
ECIRyC Total	31.47 (9.68)	8–60
DTCQ Positive Situations	73.95 (23.76)	4–100
DTCQ Negative Situations	68.53 (25.77)	4.76–100
DTCQ Temptation Situations	62.01 (29.85)	1.11–100

**Table 2:** Pearson correlation coefficients among study variables (*N =* 122).

	1	2	3	4	5	6	7	8
1. Years of sustained addiction	-							
2. Number of previous treatments	0.21 *	-						
3. SDQ Manipulation	0.12	−0.06	-					
4. SDQ Mystification	0.03	0.06	0.51 **	-				
5. ECIRyC Total	−0.03	−0.03	0.49 **	0.41 **	-			
6. DTCQ Positive Situations	0.11	−0.03	0.16	0.12	0.13	-		
7. DTCQ Negative Situations	0.01	0.06	−0.23 **	−0.20 *	−0.24 **	0.05	-	
8. DTCQ Temptation Situations	0.02	−0.04	−0.30 **	−0.23 *	−0.24 **	0.07	0.83 **	-

Note. ** *p* < 0.01; * *p* < 0.05; SDQ = Self-Deception Questionnaire; ECIRyC = Impulsive Control Scale Ramón y Cajal; DTCQ = Drug Taking Confidence Questionnaire.

**Table 3:** Stepwise Regression Model on Self-Deception Variables.

Dependent Variable	Model	Predictors	*B*	*SD*	*β*	*t*	*p*	95% CI for *B*		Adjusted *R*^2^	∆ *R*^2^
								Lower	Upper		
SDQ Manipulation ^a^	1	ECIRyC	0.325	0.054	0.485	6.03	<0.001	0.218	0.431	0.23	0.23 **
	2	ECIRyC	0.294	0.054	0.440	5.41	<0.001	0.186	0.402	0.26	0.034 *
		DTCQ Temptation Situations	−0.003	0.001	−0.191	−2.35	0.02	−0.005	−0.001		
	3	ECIRyC	0.297	0.054	0.444	5.52	<0.001	0.190	0.403	0.28	0.025 *
		DTCQ Temptation Situations	−0.003	0.001	−0.192	−2.39	0.02	−0.005	−0.001		
		Years of addiction	0.090	0.046	0.155	1.99	0.04	0.001	0.181		
SDQ Mystification ^b^	1	ECIRyC	0.275	0.056	0.410	4.89	<0.001	0.164	0.387	0.17	0.17 **

Note. ** *p* < 0.01; * *p* < 0.05; SDQ = Self-Deception Questionnaire; ECIRyC = Impulsive Control Scale Ramón y Cajal; DTCQ = Drug Taking Confidence Questionnaire; ^a^ excluded variables: number of previous treatments, DTCQ Positive Situations and DTCQ Negative Situations; ^b^ excluded variables: years of addiction, number of previous treatments, DTCQ Positive Situations and DTCQ Negative Situations and DTCQ Temptation Situations.

## Data Availability

De-identified data supporting the findings of this study are available and can be accessed through the following link: https://data.mendeley.com/drafts/m68fc6g6yy (accessed on 29 January 2025). All materials used in the study are available upon request from the authors due to copyright restrictions.

## Abbreviations

The following abbreviations are used in this manuscript:

SUD	Substance Use Disorder
SDQ-12	Self-Deception Questionnaire
ECIRyC	Impulsive Control Scale Ramón y Cajal
DTCQ	Drug Taking Confidence Questionnaire
